# Mr. Phythian on Medical Electricity

**Published:** 1812-06

**Authors:** John Phythian

**Affiliations:** Surgeon. Winchcomb


### *To the Editors of the Medical and Physical Journal.*

GENTLEMEN,

HAVING been in extensive practice for more than twenty years, I have, in many severe cases, with great pleasure observed the good effects of electricity *properly* conducted; and have long lamented that medical gentlemen have paid so little attention to this powerful agent. I was therefore much pleased with the judicious observations on the subject in the Med. Jour. vol. xxvi. fol. 109, and have

 anxiously anxiously waited for an answer to the question respecting the most advantageous form for an electrical machine, &c. which in my opinion is of great importance. The machine I have used is Nairns; but I know many gentlemen prefer the plate-form. I shall therefore feel myself greatly obliged, if, through the medium of your Journal, some gentleman, competent to the subject, will have the goodness to give his opinion respecting the above question.

I am, Gentlemen,

*Winchcomb,* Your most obedient Servant,

*March 7th,* 1812. JOHN PHYTHIAN, Surgeon.

